# Rivaroxaban plasma levels in patients admitted for bleeding events: insights from a prospective study

**DOI:** 10.1186/s12959-018-0183-3

**Published:** 2018-11-12

**Authors:** Anne-Laure Sennesael, Anne-Sophie Larock, Jonathan Douxfils, Laure Elens, Gabriel Stillemans, Martin Wiesen, Max Taubert, Jean-Michel Dogné, Anne Spinewine, François Mullier

**Affiliations:** 1 Louvain Drug Research Institute, Clinical Pharmacy Research Group, Université catholique de Louvain, Brussels, Belgium; 2 CHU UCL Namur, Namur Thrombosis and Hemostasis Center, Department of Pharmacy, Université catholique de Louvain, Yvoir, Belgium; 3 Namur Research Institute for LIfe Sciences, Namur Thrombosis and Hemostasis Center, Department of Pharmacy, University of Namur, Namur, Belgium; 4 Louvain Drug Research Institute, Integrated PharmacoMetrics, PharmacoGenomics and PharmacoKinetics, Université catholique de Louvain, Brussels, Belgium; 5 Center of Pharmacology, Therapeutic Drug Monitoring Unit, University Hospital of Cologne, Cologne, Germany; 6 Center of Pharmacology, Clinical Pharmacology Unit, University Hospital of Cologne, Cologne, Germany; 7 CHU UCL Namur, Namur Thrombosis and Hemostasis Center, Hematology Laboratory, Université catholique de Louvain, Yvoir, Belgium

**Keywords:** Direct oral anticoagulants, Rivaroxaban, Bleeding, Drug monitoring, Pharmacogenomics, Patient safety

## Abstract

**Background:**

Serious bleeding events have been frequently described in patients taking direct oral anticoagulants (DOAC). In secondary analyses of phase 3 trials, DOAC plasma concentrations were shown to correlate with bleeding outcomes. This study aimed to describe rivaroxaban plasma levels in patients admitted to the emergency department (ED) for bleeding events. For each patient, risk factors for experiencing bleeding events were also investigated.

**Methods:**

This analysis was part of an observational study conducted in the ED of two teaching hospitals. Plasma samples from 10 rivaroxaban-treated patients admitted for bleeding events were collected. Rivaroxaban plasma concentrations were determined by calibrated chromogenic anti-Xa assay. The measured rivaroxaban levels were then extrapolated at trough using a published population pharmacokinetic (PopPK) model, and compared to on-therapy ranges observed in large clinical trials. For each patient, clinical, medication and *ABCB1* genotype data were collected.

**Results:**

Rivaroxaban measurements varied from 5 to 358 ng/ml, with a post-intake delay ranging from 9 to 38 h. At trough, estimated plasma concentrations were between 12 and 251 ng/ml (median value 94 ng/ml). Four patients had higher-than-expected rivaroxaban levels. Inadequate dose regimen, excessive alcohol consumption and lack of treatment reassessment were observed in several patients. Half of patients were taking ≥1 drug with potential pharmacokinetics interactions (e.g. amiodarone, diltiazem), while half of patients were taking ≥1 drug increasing the risk of bleeding. All 3 patients with available genotyping data and higher-than-expected rivaroxaban levels were heterozygous or homozygous mutated for the *ABCB1* 1236C > T, 2677G > T, 3435 C > T and rs4148738 single nucleotide polymorphisms (SNP).

**Conclusions:**

Rivaroxaban patients admitted to the ED for bleeding events showed highly variable plasma concentrations. This analysis underlines the usefulness of rapid DOAC measurement and the value of PopPK models to estimate concentrations at trough in a context where the post-intake delay is unmanageable. Close patient follow-up, including renal function assessment and drug interactions review, is essential for bleeding risk minimization.

## Background

Direct oral anticoagulants (DOAC) are increasingly used in clinical practice, to prevent venous thrombosis or thrombus formation in non-valvular atrial fibrillation [1, 2]. However, serious bleeding events have been frequently described for patients taking DOACs. In 2013–2014, rivaroxaban and dabigatran were among the drugs most commonly implicated in emergency department (ED) admissions in the United States [3]. During phase 3 trials, risk of major bleeding of DOACs was similar to or lower than warfarin, whereas the risk of intracranial hemorrhage was substantially reduced [4, 5]. Recent observational studies have shown similar results in a real-life setting [6].

Although DOACs do not require close therapeutic monitoring, their measurement remains useful in specific clinical situations such as major bleeding and emergency surgery [7]. Assessment of the individual response may also benefit patients with suspected drug accumulation or therapeutic failure. In secondary analyses of phase 3 trials, DOAC plasma concentrations were shown to correlate with bleeding outcomes [8–10]. Conversely, a recent observational study has highlighted the relationship between low DOAC levels, measured in the first month of treatment, and the occurrence of thromboembolic events [11]. However, a therapeutic range has not been defined yet for each DOAC.

To our knowledge, few studies have investigated the intensity of DOAC anticoagulation in patients presenting to the ED for bleeding events. In the RE-VERSE AD study, the median (unbound) dabigatran plasma concentration was 110 ng/ml in patients with uncontrollable or life-threatening bleeding, before the administration of idarucizumab [12]. Bouget and Oger described DOAC levels in 5 patients admitted for major bleeding [13]. However, measurements were not interpreted by taking into account the post-intake delay and other influencing individual factors.

In this study, we aimed to describe rivaroxaban levels in patients admitted to the ED for bleeding events. Measured plasma concentrations were extrapolated at trough using population pharmacokinetic (popPK) modeling, and compared to the expected on-therapy range. Several determinants of bleeding were previously reported in rivaroxaban-treated patients, including older age, renal impairment or drug interactions [14–17]. The inappropriate use of DOAC has also been highlighted in this respect [18, 19]. In addition, *ABCB1* genetic polymorphisms have recently been suggested to contribute to high rivaroxaban levels in a patient admitted for gastrointestinal bleeding [20]. Therefore, a second objective was to investigate and discuss the presence of such risk factors for bleeding events in our rivaroxaban patients.

## Methods

### Setting and population

This analysis was part of a prospective observational cohort study, conducted in two teaching hospitals in Belgium to assess the preventability of serious adverse drug reactions related to the use of oral anticoagulants [21]. We collected plasma samples from rivaroxaban patients admitted to the ED for a bleeding event, between July 2015 and January 2016.

### Rivaroxaban measurement

Blood sampling was performed closely after management and inclusion of the patient in the study. Blood was taken by antecubital venipuncture and collected into 0.109 M sodium citrate (9:1 *v*/v) tubes. Platelet-poor plasma (PPP) was obtained from the supernatant fraction after double centrifugation for 15 min at 1500 g at room temperature. PPP was next aliquoted and frozen at − 80 °C without delay. Frozen plasma samples aliquots were thawed by heating to 37 °C for at least 5 min just before experiments.

The following routine clotting assay was performed: prothrombin time (PT) using RecombiPlasTin2G® (Werfen, Lexington, USA; normal range 12.4–14.5 s). Rivaroxaban plasma concentrations were estimated with the Biophen® Direct Factor Xa Inhibitors (Biophen®DiXaI, Hyphen BioMed, Neuville-Sur-Oise, France), a calibrated chromogenic anti-Xa assay. The procedure was performed on a STA-R® Evolution analyzer according to the manufacturer recommendations, using calibrators from Hyphen BioMed. Commercial rivaroxaban anti-Xa assays have previously demonstrated good accuracy (bias below 8%) and acceptable precision (inter-laboratory coefficients of variation (CV) 6–25%) [22]. A procedure dedicated to low concentrations was applied to rivaroxaban plasma concentrations < 50 ng/ml, where plasma samples were diluted 1:8 in buffer and low concentrations standards were used [23].

We extrapolated rivaroxaban plasma concentrations at trough (i.e. 12 h and 24 h after the last drug intake for twice daily and once daily regimen, respectively). To this end, we used a Pop PK model that was previously developed to predict adequate discontinuation intervals in a cohort of rivaroxaban patients scheduled for cardiac catheterization [24]. Residual rivaroxaban concentrations were described by a 2-compartment model with first-order absorption and elimination. Results were then compared to the expected on-therapy ranges at trough (5th–95th percentiles): 12–137 ng/ml (rivaroxaban 20 mg) and 18–136 ng/ml (rivaroxaban 15 mg) for stroke prevention in non-valvular atrial fibrillation (NVAF), and 6–87 ng/ml for treatment and secondary prevention of venous thromboembolism (VTE) [25].

### Data collection

A comprehensive medication history was performed with patients and/or relatives to collect sociodemographic, clinical and medication data (including dose regimen, indication and timing of the last rivaroxaban intake). Patient renal function was estimated based on serum creatinine on admission using Cockroft-Gault equation. We reviewed electronic medical records and contacted GPs, community pharmacists or relatives when necessary. Bleeding severity was assessed using the International Society on Thrombosis and Haemostasis definition. Patient follow-up was undertaken to assess 3-month mortality.

Appropriateness of rivaroxaban prescribing was analyzed according to the Medication Appropriateness Index (MAI) [26]. We applied a DOAC-specific form, previously developed in a pilot study [27]. We documented the concomitant use of P-glycoprotein (P-gp) and CYP3A4 inhibitors or inducers. Regarding potential pharmacodynamics interactions, we considered other anticoagulants, antiplatelet therapy, non-steroidal anti-inflammatory drugs (NSAID), selective serotonin reuptake inhibitors (SSRI) and serotonin-norepinephrine reuptake inhibitors (SNRI). Patient adherence to anticoagulant therapy was evaluated during medication history using an adapted version of the Morisky Medication Adherence Scale [28].

### *ABCB1* genetic polymorphisms

For all patients except one (because of misunderstanding), an additional blood sample was drawn in EDTA tube and frozen at − 20 °C without delay until experiments. Genomic DNA was extracted from whole blood, using QIAamp DNA Blood Mini kit (Qiagen, CA, USA). The following single nucleotide polymorphisms (SNP) of *ABCB1* were detected: rs1128503 (1236C > T, Gly412Gly), rs2032582 (2677G > T, Ala893Ser), rs1045642 (3435 C > T, Ile1145Ile) and rs4148738. Allelic discrimination was performed by TaqMan® (Applied Biosystems, CA, USA) Drug Metabolism Genotyping Assays (C___7586662_10, C_11711720C_30, C__15951365_20, C___1253813_10). Statistical analyses were performed using JMP Pro 13.2.0 (SAS Institute, Cary, NC, USA). Kruskal-Wallis tests were carried out to compare estimated trough concentrations between different genotypes. A *p*-value of less than 0.05 was considered statistically significant.

## Results

### Study population

Plasma samples from 10 rivaroxaban patients were included in this analysis. Median age of patients was 75 years, and 80% were female. Five patients had moderate renal impairment on admission (creatinine clearance < 60 ml/min). Indications of rivaroxaban therapy were stroke prevention in NVAF (6/10) and treatment and secondary prevention of VTE (4/10). Clinical characteristics are detailed in Table 1 for each patient. The most frequent adverse events were gastrointestinal bleeding (4/10). Half of bleeding events were considered major and half occurred spontaneously. Red blood cells transfusion and prothrombin complex concentrates administration were operated in 4 and 1 patients, respectively. All patients were alive at 3-month follow-up. Details of bleeding events are presented in Table 2.

**Table 1:** Characteristics of rivaroxaban patients

No	Sex, age (years)	Weight (kg)	BMI (kg/m^2^**)**	CLcr (ml/min)	Hb (g/dl)	Indication	Dosage	Duration	CHA_2_DS_2_-VASC	HAS-BLED
1	M, 83	79	23.1	58	13.7	SPAF	20 mg OD	≤ 1 year	5	3
2	F, 70	N/K	N/K	N/K	3.1	SPAF	15 mg OD	> 1 year	3	1
3	F, 87	65	25.7	48	6.4	VTE	20 mg OD	< 30 days	N/A	2
4	F, 67	80	24.7	53	7.2	SPAF	20 mg OD	> 1 year	6	2
5	F, 77	63	25.2	61	14.3	SPAF	15 mg OD	> 1 year	7	3
6	M, 66	100	35	86	15.6	SPAF	20 mg OD	≤ 1 year	2	2
7	F, 72	66	21.6	46	12.8	VTE	20 mg OD	> 1 year	N/A	3
8	F, 90	70	N/K	60	13.8	SPAF	15 mg OD	N/K	6	1
9	F, 86	89	31.2	57	14.9	VTE	15 mg BID	< 30 days	N/A	1
10	F, 69	73	26.8	76	11.3	VTE	20 mg OD	> 1 year	N/A	2

*BID* twice-daily, *BMI* body mass index, *CLcr* creatinine clearance (Cockroft-Gault equation), *Hb* haemoglobin, *OD* once-daily, *N/A* not applicable, *N/K* not known, *SPAF* stroke prevention in atrial fibrillation, *VTE* venous thromboembolism

**Table 2:** Characteristics, management and clinical outcomes of the 10 bleeding events

No	Site of bleeding	Bleeding severity	Bleeding occurrence	Management	Length of stay (days)	90-day outcome
1	Hematuria	NMCR	Trauma	–	0	Alive
2	Gastrointestinal	Major	Spontaneous	RBC (3 units)^a^	N/K	N/K
3	Gastrointestinal	Major	Spontaneous	RBC (2 units)^a^	3	Alive
4	Gastrointestinal	Major	Spontaneous	RBC (3 units)^a^	1	Alive
5	Intracranial	Major	Trauma	PCC (2500 IU)	43	Alive
6	Gastrointestinal	NMCR	Post-intervention	–	0	Alive
7	Epistaxis	NMCR	Spontaneous	–	2	Alive
8	Intracranial	Major	Trauma	–	1	Alive
9	Hematuria	NMCR	Spontaneous	–	0	Alive
10	Hematoma	NMCR	Trauma	–	0	N/K

*N/K* not known, *NMCR* non-major clinically relevant, *PCC* prothrombin complex concentrates, *RBC* red blood cells. ^a^Transfusion before blood sampling

### Rivaroxaban measurement

Measured rivaroxaban levels varied from 5 to 358 ng/ml, with a delay since the last drug intake ranging from 9 to 38 h. PT was above the normal range in all patients with rivaroxaban concentrations above 30 ng/ml (Table 3). When using PopPK model, estimated plasma concentrations at trough were between 12 and 251 ng/ml (median value 94 ng/ml). Elimination clearance (CL/F) was between 2.0 and 7.9 L/h (median value 3.3 L/h). Measured and extrapolated rivaroxaban concentrations are described in Table 3 for each patient, as well as the elimination clearance. Four patients had rivaroxaban levels at trough higher than the expected on-therapy range (No 2, 3, 5, 9).

**Table 3:** Measurement of rivaroxaban plasma concentrations

No	Delay (h)	PT (sec)	Biophen® DiXaI (ng/ml)	Estimated cc at trough (ng/ml)	Clearance (L/h)	Potential PK drug interactions	Potential PD drug interactions
1	28	18.7	84.8	98	3.3	–	Ibuprofen
2	22	19.6	237.5	**181** ^a^	2.0	Diltiazem (↑), Clarithromycin (↑)	Escitalopram
3	38	15.4	70.8	**125** ^a^	2.9	Simvastatin (↑)	/
4	27	17.7	58.3	69	3.9	–	Diclofenac, Escitalopram
5	27	N/K	139.4	**134** ^a^	2.4	Amiodarone (↑)	Aspirin
6	26	12.9	4.9	12	7.9	Amiodarone (↑)	/
7	37	14.4	18.3	44	4.8	Diltiazem (↑)	/
8	27	15.3	86.5	89	3.0	/	/
9	9	26.0	357.8	**251** ^a^	3.2	/	/
10	13	N/K	184.7	72	3.9	/	Duloxetine, Paroxetine

*Biophen®DiXaI* Biophen® Direct Factor Xa Inhibitors, *cc* concentration, *N/K* not known, *PD* pharmacodynamics, *PK* pharmacokinetics, *PT* prothrombin time (normal range: 12.4–14.5 s), ↑: increased plasma concentrations, ↓: decreased plasma concentrations

^a^**Estimated trough concentration above the on-therapy range (**we considered higher-than-expected rivaroxaban levels for patient 5, as measured concentration was higher than 136 ng/ml)

### Analysis of risk factors for bleeding events

The analysis of rivaroxaban prescriptions revealed that at least one criterion of the MAI was inappropriate in 8 of 10 patients. Three patients received an inadequate dosing regimen: 20 mg OD instead of 15 mg OD (No 3, 7), and 15 mg BID instead of 20 mg OD (No 9). Two of them had estimated trough concentration above the on-therapy range. Three patients had an excessive alcohol consumption (≥ 2 units/day; No 1, 4, 7). Moreover, in two patients treated for VTE prevention, treatment indication had not been reassessed for years (No 3, 10).

Half of patients were taking at least 1 drug with potential pharmacokinetic interactions (Table 3). Three of them had higher-than-expected rivaroxaban plasma concentrations at trough. The most frequent interacting drugs were amiodarone and diltiazem. Moreover, half of patients were taking at least 1 drug increasing the risk of bleeding (Table 3). We mainly observed the concomitant use of SSRI (*n* = 3) and NSAID (*n* = 2). Regarding adherence to anticoagulant therapy, three patients reported forgetting to take their medicine in rare circumstances (No 1, 4, 7). One patient mentioned a variable time of intake (No 7).

*ABCB1* genetic polymorphisms were analyzed in 9 of 10 patients. Genotyping data are presented in Table 4 for the *ABCB1* 1236C > T, 2677G > T, 3435 C > T and rs4148738 SNP. Of the four patients with higher-than-expected rivaroxaban trough levels, three carried at least one variant allele for each of these SNP (No 2, 3, 5 – blood sample not available for patient No 9). However, no clear association between ABCB1 genotype and estimated trough concentrations was observed (*p* > 0.05).

**Table 4:** *ABCB1* genotyping in 10 rivaroxaban patients with bleeding events

No	1236C > T	2677G > T	3435C > T	rs4148738
1	CC	GG	CC	AA
2	**CT**	**GT**	**CT**	**GA**
3	**CT**	**GT**	**CT**	**GA**
4	TT	TT	CT	GG
5	**CT**	**GT**	**TT**	**GA**
6	TT	TT	TT	GG
7	CT	GT	TT	GA
8	TT	TT	TT	GG
9	**N/K**	**N/K**	**N/K**	**N/K**
10	CC	GG	CT	AA

*N/K* not known. Patients with higher-than-expected rivaroxaban levels are shown in bold

## Discussion

In this analysis of 10 patients admitted for bleeding events, we observed a large range of rivaroxaban levels. The application of a previously published PopPK model revealed that four patients had higher-than-expected plasma concentrations at trough. Several risk factors for bleeding were found at the individual level, including older age, inappropriate use of rivaroxaban and drug interactions.

To our knowledge, this is the first study to analyze in depth rivaroxaban measurements in the context of bleeding events. Inter-individual variability in DOAC exposure has been previously investigated in routine care, showing plasma levels outside the on-therapy range in 40% of rivaroxaban patients [29]. In four Italian anticoagulation clinics, rivaroxaban measurements varied nearly 15-fold among NVAF patients, with a mean trough level around 40 ng/ml [30]. This variability is in agreement with our results, although we estimated a higher median trough concentration (94 ng/ml). This reflects a delayed rivaroxaban clearance in our bleeding patients (median CL/F 3.3 L/h, compared to 4.9 L/h in patients scheduled for cardiac catheterization or 6.1 L/h in AF patients as previously reported) [24, 31]. Two recent cohort studies have shown median rivaroxaban levels of 124 ng/ml in patients with severe bleeding events, and 102 ng/ml in patients admitted for intracranial hemorrhage [32, 33]. Delay since the last drug intake was 12 h (median value) in the former case, and less than 24 h for most patients in the latter case.

### Rivaroxaban measurement for bleeding management

The management of DOAC-related bleeding includes the temporary discontinuation of the oral anticoagulant, supportive measures and the administration of reversal agents, depending on the severity of the event [34, 35]. Andexanet alpha has recently been approved by the FDA to reverse the anticoagulant effect of rivaroxaban, while prothrombin complex concentrates were also suggested as an alternative [36–38]. In this context, rapid laboratory measurement may provide valuable assistance to warrant and monitor antidote administration, or manage urgent interventions [7].

In the present study, we observed that PT was prolonged in all patients with clinically relevant concentrations (> 30 ng/ml) of rivaroxaban. However, we employed a sensitive thromboplastin reagent (RecombiPlasTin 2G®) for the assay [39]. A nationwide Belgian survey has previously highlighted a wide variation in response to rivaroxaban according to the reagent used [40]. Commercial specific assays are currently available for all DOACs [41]. They are more accurate but require calibrators and controls. Turnaround times around 30 min have nevertheless been reported in a daily practice context [42, 43].

### Factors associated with an increased risk of bleeding

Several risk factors for bleeding events were highlighted in our 10 rivaroxaban patients. First, half of them were older than 75 years, and half of them had moderate renal impairment. Older age and renal insufficiency were previously demonstrated as contributing factors to major bleeding in rivaroxaban patients [14]. Recently, glomerular filtration rates below 60 ml/min have been independently associated with higher-than-expected residual rivaroxaban levels in a perioperative setting [44]. This is not surprising since one third of the rivaroxaban dose is eliminated unchanged by the kidneys [25]. However, in our analysis, only two patients with moderate renal impairment had trough concentrations above the on-therapy range. Second, despite the highest convenience and fixed-dose regimen of DOAC, some of our patients did not receive the appropriate dose of rivaroxaban. In particular, one patient (No 9) was still taking rivaroxaban 15 mg BID while the 21-day treatment phase of VTE had been completed. Yao and colleagues highlighted that patients with indication for dose reduction were often potentially overdosed, leading to an increased risk of major bleeding [19].

Drugs interactions with P-gp or CYP3A4 inhibitors were frequent in our patients, as previously reported [32, 45]. One patient (No 2) was taking diltiazem and clarithromycin, two inhibitors of both P-gp and CYP3A4. He had an estimated rivaroxaban level of 181 ng/ml at trough, exceeding the 95th percentile of the on-therapy range (136 ng/ml). In healthy volunteers, clarithromycin has been shown to promote a 2-fold increase in rivaroxaban exposure [46]. Similarly, we extrapolated a rivaroxaban trough concentration of 125 ng/ml in a patient taking simvastatin (No 3). This P-gp inhibitor has been associated with a higher risk of major bleeding in patients taking dabigatran [47]. Pharmacodynamic interactions were also widely observed. Aspirin has often no valid indication in anticoagulated patients, while combination therapy has been shown to increase the risk of major bleeding [16, 48]. A similar increase was associated with the addition of SSRIs, the most prescribed class of antidepressants [49].

An original aspect of this work was the investigation of several *ABCB1* polymorphisms. All 3 patients with higher-than-expected rivaroxaban levels and available genotyping data were at least heterozygous mutated for the 1236C > T, 2677G > T, 3435C > T and rs4148738 SNP. Previous experiments have shown that the 1236 T–2677 T-3435 T variant haplotype decreased ABCB1 expression or transport towards several drugs such as anticancer agents [50–52]. These results support our observations, as a decreased efflux of rivaroxaban may have led to drug accumulation. However, in a recent study conducted in 60 healthy volunteers, the 1236 T–2677 T-3435 T haplotype was not a significant determinant of rivaroxaban pharmacokinetics [46]. This strengthens the need for additional studies to clarify the impact of *ABCB1* polymorphisms on rivaroxaban transport. Indeed, our findings might also be due to the high frequency of the variant genotype 1236 T–2677 T-3435 T (up to 50% of the Caucasian population is expected to heterozygous mutated). Furthermore, these three patients were also receiving concomitant interacting medications, as previously discussed.

The study presents several limitations. First, statistical analysis was limited by the small number of plasma samples collected. However, this allowed an in-depth analysis of rivaroxaban measurements, including clinical, medication and genetic characteristics at the individual level. Second, three patients (No 2–4) were transfused with red blood cells (RBC) before sampling. For these patients, we cannot exclude that rivaroxaban measurements were not influenced by fluid volume or factor Xa content of packed RBC. However, Biophen®DiXaI was designed for minimizing the interference of plasma factors. Third, estimation of renal function was only based on serum creatinine on admission, as previous laboratory results performed in primary care were not available. Finally, the Pop PK model we used assumed no variability in the volume of distribution, while inter-individual variability in V/F was 18% in another Pop PK model from AF patients [31]. However, simulations were previously performed and showed the limited influence of this variability on predicted exposure estimates.

## Conclusion

In conclusion, rivaroxaban patients admitted to the ED for bleeding events showed highly variable plasma concentrations and a delayed elimination clearance. This underlines the usefulness of rapid DOAC measurement to assess contribution to the adverse event, manage urgent procedures or warrant antidote administration. Close patient follow-up, including renal function assessment and drug interactions review, is essential for bleeding risk minimization. Further studies are needed to investigate the impact of *ABCB1* polymorphisms on DOAC transport.
